# Impact of signal intensity normalization of MRI on the generalizability of radiomic-based prediction of molecular glioma subtypes

**DOI:** 10.1007/s00330-023-10034-2

**Published:** 2023-09-06

**Authors:** Martha Foltyn-Dumitru, Marianne Schell, Aditya Rastogi, Felix Sahm, Tobias Kessler, Wolfgang Wick, Martin Bendszus, Gianluca Brugnara, Philipp Vollmuth

**Affiliations:** 1 Department of Neuroradiology, Heidelberg University Hospital, Heidelberg, DE Germany; 2 Section for Computational Neuroimaging, Department of Neuroradiology, Heidelberg University Hospital, Heidelberg, DE Germany; 3 Department of Neuropathology, Heidelberg University Hospital, Heidelberg, DE Germany; 4 Department of Neurology, Heidelberg University Hospital, Heidelberg, DE Germany; 5 Clinical Cooperation Unit Neurooncology, German Cancer Research Center (DKFZ), Heidelberg, DE Germany; 6 Division of Medical Image Computing (MIC), German Cancer Research Center (DFKZ), Heidelberg, Germany

**Keywords:** Glioma, Magnetic resonance imaging, Genotype, Isocitrate dehydrogenase

## Abstract

**Objectives:**

Radiomic features have demonstrated encouraging results for non-invasive detection of molecular biomarkers, but the lack of guidelines for pre-processing MRI-data has led to poor generalizability. Here, we assessed the influence of different MRI-intensity normalization techniques on the performance of radiomics-based models for predicting molecular glioma subtypes.

**Methods:**

Preoperative MRI-data from *n* = 615 patients with newly diagnosed glioma and known isocitrate dehydrogenase (IDH) and 1p/19q status were pre-processed using four different methods: no normalization (naive), N4 bias field correction (N4), N4 followed by either WhiteStripe (N4/WS), or *z*-score normalization (N4/*z*-score). A total of 377 Image-Biomarker-Standardisation-Initiative-compliant radiomic features were extracted from each normalized data, and 9 different machine-learning algorithms were trained for multiclass prediction of molecular glioma subtypes (IDH-mutant 1p/19q codeleted vs. IDH-mutant 1p/19q non-codeleted vs. IDH wild type). External testing was performed in public glioma datasets from UCSF (*n* = 410) and TCGA (*n* = 160).

**Results:**

Support vector machine yielded the best performance with macro-average AUCs of 0.84 (naive), 0.84 (N4), 0.87 (N4/WS), and 0.87 (N4/*z*-score) in the internal test set. Both N4/WS and *z*-score outperformed the other approaches in the external UCSF and TCGA test sets with macro-average AUCs ranging from 0.85 to 0.87, replicating the performance of the internal test set, in contrast to macro-average AUCs ranging from 0.19 to 0.45 for naive and 0.26 to 0.52 for N4 alone.

**Conclusion:**

Intensity normalization of MRI data is essential for the generalizability of radiomic-based machine-learning models. Specifically, both N4/WS and N4/*z*-score approaches allow to preserve the high model performance, yielding generalizable performance when applying the developed radiomic-based machine-learning model in an external heterogeneous, multi-institutional setting.

**Clinical relevance statement:**

Intensity normalization such as N4/WS or N4/*z*-score can be used to develop reliable radiomics-based machine learning models from heterogeneous multicentre MRI datasets and provide non-invasive prediction of glioma subtypes.

**Key Points:**

*• MRI-intensity normalization increases the stability of radiomics-based models and leads to better generalizability.*

*• Intensity normalization did not appear relevant when the developed model was applied to homogeneous data from the same institution.*

*• Radiomic-based machine learning algorithms are a promising approach for simultaneous classification of IDH and 1p/19q status of glioma.*

**Supplementary information:**

The online version contains supplementary material available at 10.1007/s00330-023-10034-2.

## Introduction

Gliomas are the most common malignant primary tumors of the central nervous system in adults and are currently stratified based on both histology and molecular markers according to the 2021 WHO Classification, where the IDH status and the 1p19q codeletion currently represent the most relevant mutations [1, 2].

There have been numerous attempts to find non-invasive radiological markers for these mutations since their inclusion in the WHO classification, both through purely visual assessment techniques [3] and through advanced imaging sequences such as perfusion-weighted or diffusion-weighted imaging with varying results [4, 5]. Radiomic features have recently demonstrated a convincing performance for non-invasive detection of mutations [6–10] but have also come under scrutiny due to a lack of reporting standards [11, 12].

This is particularly relevant for MRI-based features, since voxel values are not based on a standardized scale but rather on arbitrary intensity values that can vary based on the device manufacturer, field strength, sequence acquisition parameters, and type, thus differing not only between patients but also between examinations of the same patient [13]. Understandably, this can affect the extraction of radiomics and hinder the reproducibility and/or comparability of results, and intensity normalization is a necessary step according to the TRIPOD/RQS Guidelines [11, 12].

Previous studies have focused on assessing the impact of various pre-processing steps onto the signal intensity and the resulting radiomic features, for example, demonstrating that bias field correction reduces the inhomogeneity within a tissue [14], and that skull-stripping can remove the regions with the greatest intensity fluctuations [15]. Even though there is a consensus on the general steps required in a radiomics pre-processing pipeline [11, 16], there is still no agreement onto which specific methodology might be the most indicated for each of them [17]. Previous studies have investigated the impact of intensity normalization on the reproducibility of radiomics, but none have compared different intensity normalization approaches on the predictive power of machine learning algorithms for the detection of radiogenomic phenotypes of brain tumors [17].

In this study, we evaluated the impact of intensity normalization on multiclass models for simultaneous prediction of IDH and 1p19q status in gliomas and verified the generalizability of the models on two external, publicly available datasets from The Cancer Genome Atlas (TCGA) project and the University of California San Francisco (UCSF).

## Material and methods

The retrospective study was approved by the internal ethics committee, and the requirement to obtain informed consent was waived (S-784 2018).

Consecutive adult patients with confirmed diagnosis of glioma according to the WHO 2021 Classification and who received preoperative MRI at the Department of Neuroradiology of the Heidelberg University Hospital (Heidelberg, Germany) between 03/2009 and 07/2020 were included in the study (*n* = 621). IDH and 1p/19q status based on DNA methylation assay was available for all patients [18]. Ultimately, *n* = 3 cases were excluded due to insufficient quality of MRI images (e.g., motion artifacts, which prevented an adequate tumor segmentation) and *n* = 3 due to errors in data processing, resulting in an internal dataset of *n* = 615 patients in total. The internal dataset (HD) was acquired during routine clinical examination using a 3-T MRI machine (Magnetom Verio, Trio TIM or Skyra, Siemens Healthcare). The imaging protocol was performed according to international guidelines and included 3D T1-weighted images both before (T1) and after (cT1) administration of a bolus of 0.1 mmol/kg gadoterate meglumine (Dotarem, Guerbet), as well as axial 2D FLAIR and T2-weighted images [19]; a detailed description of the MRI acquisition parameters is available in the Supplementary Methods and Materials.

External testing was performed using two publicly available preoperative MRI datasets of glioma, namely the public dataset from TCGA database with 242 patients and the UCSF dataset with 501 patients [20, 21]. From the TCGA dataset, *n* = 41 cases were removed due to insufficient quality of MRI images, *n* = 29 cases due to unknown IDH or 1p/19q status, and *n* = 12 due to failed intensity normalization, so that the TCGA dataset ultimately comprised *n* = 160 patients. From the UCSF dataset, *n* = 91 cases were removed due to unknown 1p/19q status, ultimately including *n* = 410 patients. As in the training dataset, data in the external validation included T1-weighted images before and after contrast administration, FLAIR, and T2 weighted images. Information on the sequences included in the datasets is found at https://doi.org/10.7937/tcia.bdgf-8v37.

### Image pre-processing and tumor segmentation

Images from the HD and TCGA dataset were processed using publicly available and validated software as previously described [22, 23]. Briefly, this included (i) neural-network-based brain extraction through the HD-BET tool (https://github.com/MIC-DKFZ/HD-BET) [24], (ii) rigid registration of the image volumes to the native T1-w image using FSL (FMRIB), and (iii) automated deep-learning based tumor segmentation into the contrast-enhancing, T2-FLAIR, and necrotic components of the tumor using a variant of HD-GLIO (https://github.com/NeuroAI-HD/HD-GLIO). The segmentations were visually reviewed and, if necessary, corrected by MF (neuroradiology resident with 5 years of experience).

The UCSF dataset consisted of pre-processed sequences as well as a multicompartment tumor segmentation including enhancing tumor, and T2/FLAIR hyperintense as well as non-enhancing/necrotic tumor compartments, downloaded through https://doi.org/10.7937/tcia.bdgf-8v37 [21].

**Fig. 1:**
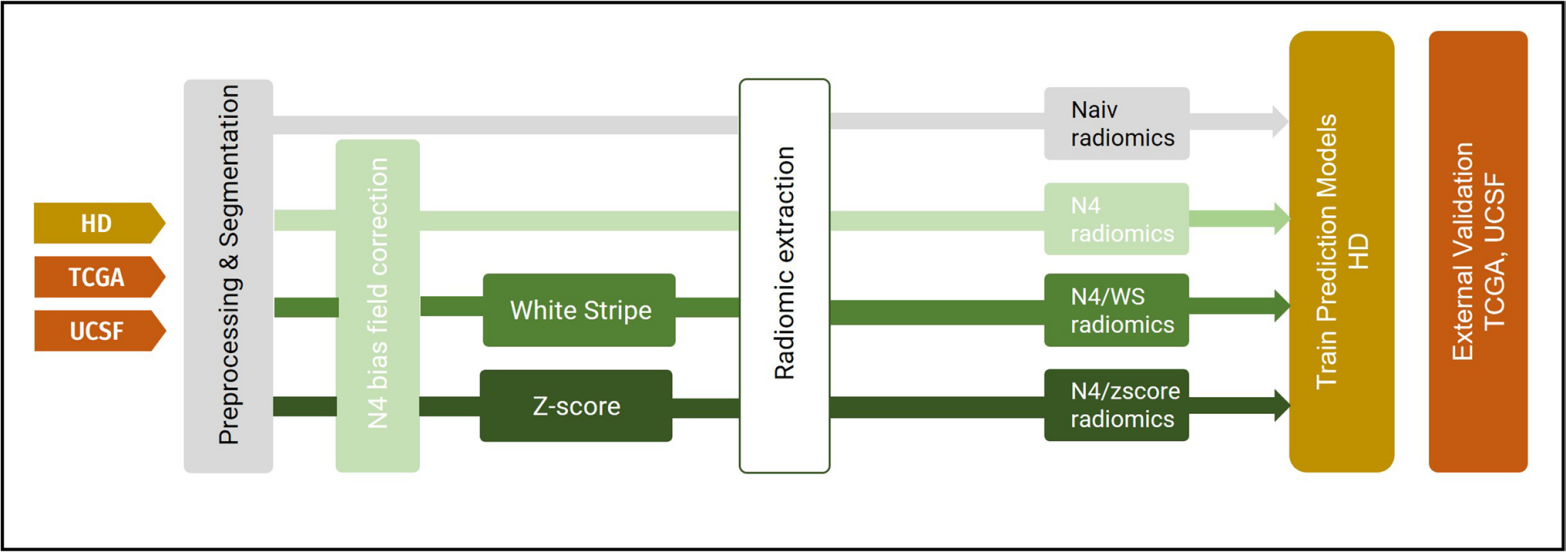
Schematic representation of the study structure. Four different conditions were compared: no normalization (naive), N4 bias field correction (N4), N4 bias field correction followed by white stripe normalization (N4/WS), and N4 bias field correction followed by *z*-score normalization (N4/*z*-score). For each of the conditions, radiomics were extracted from all four sequences (T1 before and after contrast, T2, and FLAIR) from each of the three data sets. Using the training dataset from HD (*n* = 492), 9 different machine learning models were trained for each condition and validated using the holdout test dataset (*n* = 123). External validation was performed using TCGA (*n* = 160) and UCSF data set (*n* = 410)

For each sequence, four normalization approaches were compared: (1) no normalization (naive), (2) N4 bias field correction (N4), (3) N4 bias field correction followed by WhiteStripe normalization (N4/WS), and (4) N4 bias field correction followed by *Z*-score normalization (N4/*z*-score). Figure 1 shows a schematic representation of the study structure. Detailed descriptions of the different normalization methods can be found in Supplementary Methods and Materials. Both the* Z*-score and WhiteStripe are freely available normalization methods that are commonly used in MRIs of the brain [25]. N4 bias field correction, as well as *Z*-score and WhiteStripe normalization were performed using the ANTsR and WhiteStripe packages implemented in R (R version 4.0.2., R Foundation for Statistical Computing, https://github.com/muschellij2/WhiteStripe).

### Radiomic feature extraction and selection

Feature extraction and selection was performed in Python (version 3.8.5) using PyRadiomics (https://pyradiomics.readthedocs.io) [9]. For the feature extraction the different segmentation masks of contrast-enhancing, T2-FLAIR and necrotic components of the tumor were combined into one, so the radiomics were extracted from the whole tumor. All reproducible radiomic features according to the Image Biomarker Standardisation Initiative were calculated on all used anatomical sequences. This included 13 shape-based, 17 first order, 23 Gy-level co-occurrence matrix, 16 Gy-level run length matrix, 16 Gy-level size zone matrix, 14 Gy-level dependence matrix, and 5 neighboring gray tone difference matrix. A list of all extracted radiomics is shown in Supplementary Table 1. As radiomics based on the morphology of the tumor are the same for all four sequences, shape features were only kept from the T1 pre-contrast sequence, resulting in a total of 377 radiomics for each intensity normalization approach in each dataset.

### Data analysis

The HD dataset was divided into a training and test set with 80:20 split and by maintaining a similar distribution across the three classes in both datasets.

Feature selection was performed to avoid overfitting using ANOVA F-statistics on the training data of the HD dataset. Here, an ANOVA was performed for each feature to classify the three different tumor classes, and the result were given as F-statistic. For the final models, we then used the five radiomic features with the highest F-statistic [26].

Machine learning models were built with the scikit-learn library in Python (version 3.8.5). We compared nine different algorithms, namely Logistic Regression, Linear Discriminant Analysis, k-nearest neighbor, Decision Tree, Gaussian naïve Bayes, C-Support Vector Classification, Random Forest, Extra-trees Classifier, and eXtreme Gradient Boosting. To avoid potential bias when comparing the different normalization approaches, we did not perform hyperparameter tuning like grid search, but instead used the default setting for each model.

Undersampling of the IDH-wt group and SMOTE oversampling of the IDH-mut group was performed to balance the training data set.

Models were evaluated with areas under the receiver operating characteristic curve (AUC), and sensitivity, specificity, and accuracy as target parameters (Supplementary Table 2–4). Confusion matrices matrices of various classifiers can be found in Supplementary Table 8. Confidence intervals were calculated using bootstrapping (Supplementary Fig. 4). DeLong’s test was used to compare AUC of the models. Subsequently, the *p* values were corrected for each data set individually using false discovery rate (FDR) correction. All corrected *p* values of the individual classes for each data set are shown in Supplementary Table 5–7. Kruskal–Wallis test was used to compare continuous and chi-square test was used to compare categorical data. A *p* value < 0.05 was considered significant for all analyses.

## Results

The HD dataset consists of *n* = 442 IDH wild-type (IDH-wt) gliomas, *n* = 89 IDH-mutated and 1p/19q non-codeleted (IDH-mut 1p/19q non-codel) gliomas as well as *n* = 84 IDH-mutated and 1p19q codeleted (IDH-mut 1p/19q codel) gliomas. The full characteristics of the included patients from each of the three datasets are summarized in Table 1.

**Table 1:** Demographics of HD, TCGA, and UCSF data set divided according to WHO tumor genetic classification

Parameter	Data	All classes	IDH mut + 1p/19q codeletion	IDH mut + 1p/19q non-codeletion	IDH wt
	HD	615	84	89	442
Total no. of patients	TCGA	160	17	42	101
	UCSF	410	15	84	311
	HD	44 (*p* = 0.56)	46 (*p* = 0.35)	35 (*p* = 0.16)	45 (*p* = 0.83)
Female (%)	TCGA	47	59	52	43
	UCSF	42	33	40	43
	HD	57 ± 15 (*p* = 0.015)	46 ± 14 (*p* = 0.07)	39 ± 11 (*p* = 0.60)	63 ± 12 (*p* = 0.013)
Mean age (year)	TCGA	54 ± 15	54 ± 12	39 ± 13	59 ± 13
	UCSF	56 ± 15	44 ± 14	38 ± 11	61 ± 12

**Fig. 2:**
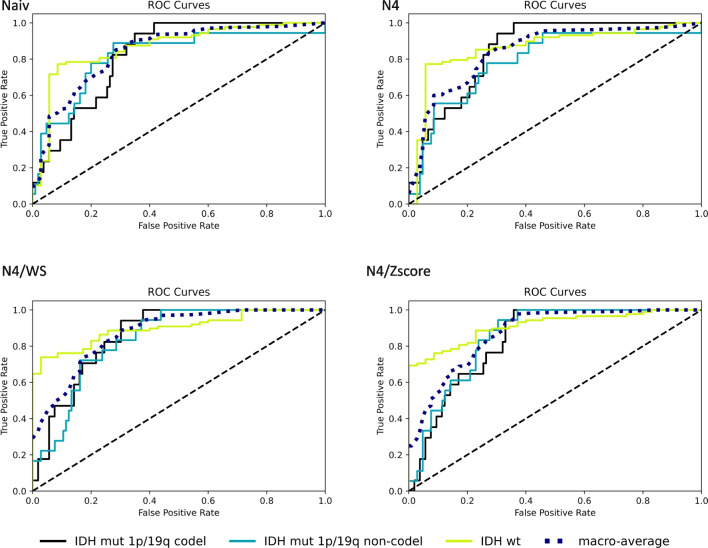
Support vector machine model performance on holdout test dataset (*n* = 123) of HD to classify IDH-wt vs. IDH-mut 1p/19q codel vs. IDH-mut 1p/19q non-codel via radiomic features across normalization conditions (Naiv, no-normalization; N4, N4 bias field correction; N4/WS, N4 bias field correction followed by white stripe normalization; and N4/*z*-score, N4 bias field correction followed by *Z*-score normalization)

We tested nine different machine learning algorithms. Among these, the support vector machine (SVM) model showed the best performance, with a macro-average AUC for the multi-class detection of molecular characteristics of 0.84 (95% CI = 0.75–0.89) using naive data, 0.84 (95% CI = 0.76–0.90) using N4, 0.87 (95% CI = 0.80–0.91) using N4/WS, and 0.87 (95% CI = 0.81–0.91) using N4/*z*-score in the HD test set. The individual detection rates for each of the molecular mutation classes were 0.87 (95% CI = 0.76–0.93) (naive), 0.86 (95% CI = 0.77–0.93) (N4), 0.90 (95% CI = 0.84–0.94) (N4/WS), and 0.91 (95% CI = 0.85–0.95) (N4/*z*-score) for the IDH-wt class, 0.82 (95% CI = 0.64–0.91) (naive), 0.79 (95% CI = 0.60–0.88) (N4), 0.84 (95% CI = 0.75–0.91) (N4/WS), and 0.86 (95% CI = 0.78–0.92) (N4/*z*-score) for the IDH-mut 1p/19q non-codel class, and 0.82 (95% CI = 0.73–0.89) (naive), 0.85 (95% CI = 0.77–0.92) (N4), 0.86 (95% CI = 0.77–0.92) (N4/WS), and 0.84 (95% CI = 0.75–0.90) (N4/z-score) for the IDH-mut 1p/19q codel class (Fig. 2), without a significant difference in model performance when comparing the different types of normalization approaches (FDR-adjusted *p* ≥ 0.19 each) (Supplementary Table 5–7). The ROC curves obtained with the eight other tested models are shown in Supplementary Fig. 1–3. The respective accuracy values including the 95% CI are shown in Supplementary Fig. 4.

**Fig. 3:**
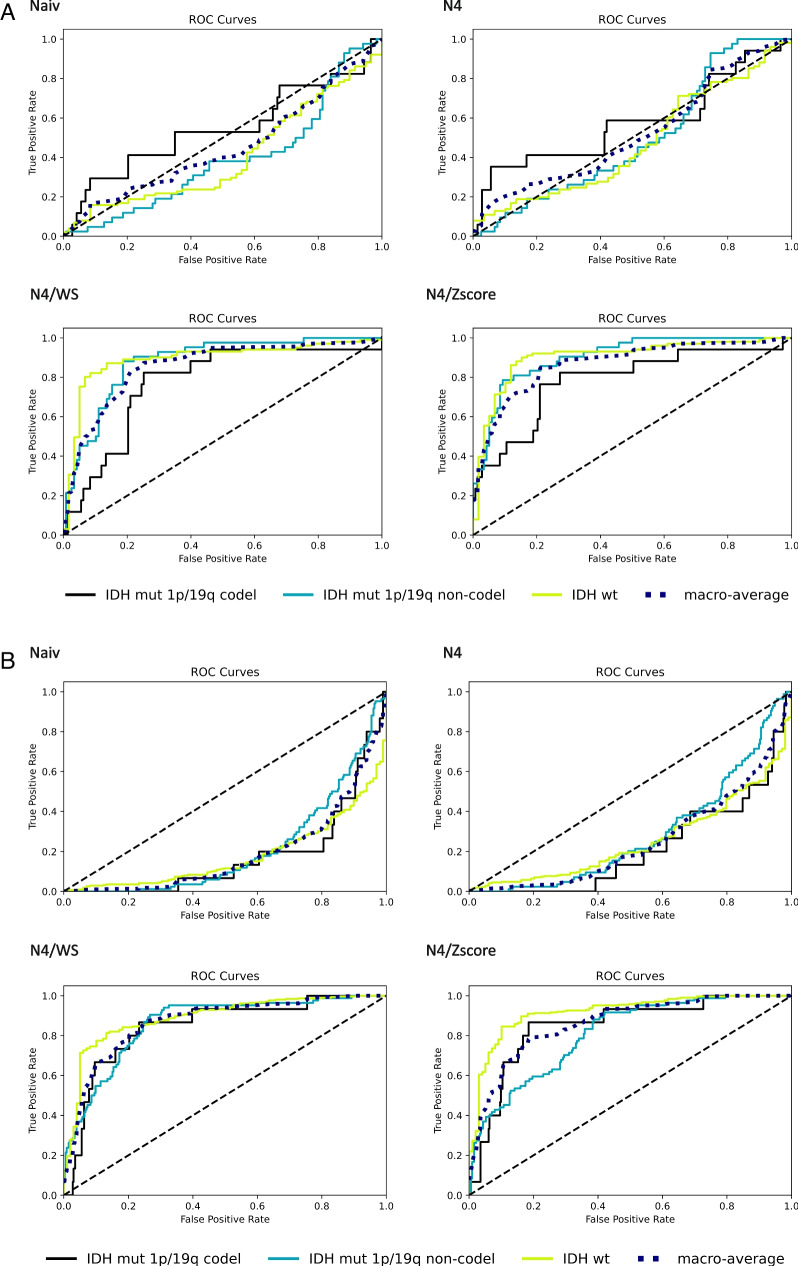
ROC curves of support vector machine model of external validation with TCGA (**A**) and UCSF data set (**B**) to differentiate between IDH-wt vs. IDH-mut 1p/19q codel vs. IDH-mut 1p/19q non-codel across different normalization conditions (Naiv, no-normalization; N4, N4 bias field correction; N4/WS, N4 bias field correction followed by white stripe normalization; and N4/*z*-score, N4 bias field correction followed by *Z*-score normalization)

When applying the developed SVM model to the external TCGA data, the macro-average AUC changed from 0.45 (95% CI = 0.35–0.54) using naive to 0.52 (95% CI = 0.43–0.61) using N4, 0.85 (95% CI = 0.76–0.89) using N4/WS, and 0.87 (95% CI = 0.80–0.91) using N4/*z*-score. The individual detection rates for each of the molecular mutation classes were 0.41 (95% CI = 0.33–0.50) (naive), 0.47 (0.38–0.56) (N4), 0.89 (0.82–0.94) (N4/WS), and 0.90 (95% CI = 0.84–0.95) (N4/*z*-score) for the IDH-wt class; 0.39 (95% CI = 0.29–0.49) (naive), 0.49 (95% CI = 0.39–0.59) (N4), 0.88 (95% CI = 0.81–0.92) (N4/WS), and 0.91 (95% CI = 0.85–0.94) (N4/*z*-score) for the IDH-mut 1p/19q non-codel class; and 0.55 (95% CI = 0.39–0.75) (naive), 0.58 (95% CI = 0.39–0.73) (N4), 0.77 (95% CI = 0.59–0.86) (N4/WS), and 0.79 (95% CI = 0.61–0.89) (N4/*z*-score) for the IDH-mut 1p/19q codel class (Fig. 3A). Both the prediction of IDH-wt and IDH-mut 1p/19q non-codel classes showed higher performance for N4/WS and N4/*z*-score as compared to approaches without normalization (FDR-adjusted *p* < 0.0001 each) as well as to N4 alone (FDR-adjusted *p* < 0.0001 each), whereas no difference was found for the IDH-mut 1p/19q codel class (FDR-adjusted *p* ≥ 0.07 each). In all three groups, there was no performance difference between N4/WS and N4/*z*-score (FDR-adjusted *p* ≥ 0.24 each).

When applying the developed SVM model to the external UCSF data, the macro-average AUC changed from 0.19 (95% CI = 0.15–0.25) using naive to 0.26 (95% CI = 0.20–0.31) using N4, 0.87 (95% CI = 0.82–0.90) using N4/WS, and 0.86 (95% CI = 0.80–0.89) using N4/*z*-score. The individual detection rates for each of the molecular mutation classes were 0.18 (95% CI = 0.14–0.23) (naive), 0.25 (95% CI = 0.20–0.31) (N4), 0.89 (95% CI = 0.84–0.92) (N4/WS), and 0.92 (95% CI = 0.87–0.94) (N4/*z*-score) for the IDH-wt class; 0.21 (95% CI = 0.16–0.26) (naive), 0.29 (95% CI = 0.23–0.34) (N4), 0.86 (95% CI = 0.81–0.90) (N4/WS), and 0.81 (95% CI = 0.76–0.86) (N4/*z*-score) for the IDH-mut 1p/19q non-codel class; and 0.18 (95% CI = 0.10–0.30) (naive), 0.22 (95% CI = 0.12–0.35) (N4), 0.84 (95% CI = 0.70–0.91) (N4/WS), and 0.85 (95% CI = 0.65–0.91) (N4/*z*-score) for the IDH-mut 1p/19q codel class (Fig. 3B). All three molecular mutation classes showed significantly higher performance for N4/WS and N4/*z*-score as compared to no normalization (FDR-adjusted *p* < 0.0001 each) as well as to N4 alone (FDR-adjusted* p* < 0.0001 each). There was no significant performance difference between N4/WS and N4/*z*-score in the IDH-mut 1p/19q codel group (FDR-adjusted *p* = 0.84). In the IDH-mut 1p/19q non-codel group the N4/WS (AUC = 0.86) outperformed the N4/*z*-score (AUC = 0.81) approach (FDR-adjusted *p* = 0.009). On the other hand, in the IDH-wt group the N4/z-score (AUC = 0.92) outperformed the N4/WS (AUC = 0.89) approach (FDR-adjusted *p* = 0.024).

## Discussion

Radiomic prediction of molecular subtypes of glioblastoma is a promising and clinically highly relevant field of research. The lack of standardized processing is negatively impacting the diffusion of radiomics by limiting their generalizability. Here, we investigated the relevance of intensity normalization as an important pre-processing step in MRI radiomics extraction and its potential influence on the predictive power of machine learning algorithms. We could demonstrate that while the model performance was not influenced by the intensity normalization of the MR images when using an internal test, however the performance depended on intensity normalization when applying the models in an external heterogeneous, multi-institutional setting. Furthermore, we were able to show that radiomic-based machine learning algorithms are a promising approach for simultaneous classification of IDH and 1p/19q status of glioma.

Our results highlight the importance of intensity normalization for removing the scanner-dependent signal intensity changes to build generalizable radiomic-based prediction models, which becomes essential when applying these models to previously unseen, heterogeneous multicenter cohorts. Although no clear consensus has emerged as to which approach is the most reliable intensity normalization approach [27], we have shown that both N4/*z*-score and N4/white-stripe yield comparable results, allowing to build generalizable radiomic-based models for predicting molecular glioma subtypes, in contrast to N4 or naive approaches which significantly limited the generalizability of these models.

In addition to the relevance of intensity normalization, we showed that radiomics-based models can perform well for simultaneous prediction of IDH and 1p/19q status in gliomas. Previous studies primarily focused on separated binary classification of IDH-mutations and/or 1p19q status in gliomas [4, 28] which may however yield inconsistent predictions not in accordance with the current WHO classification of CNS tumors (e.g., prediction of 1p19q codeletion in the setting of IDH wild-type tumors). We circumvented this by implementing a multiclass model which directly predicts the molecular glioma subtypes, i.e., classifying cases as either IDH-wt, or IDH-mut 1p19q code, or IDH-mut 1p19q non-codel. Similarly, Cluceru and colleagues previously presented a multiclass model for the simultaneous classification of IDH and 1p/19q status [7] which yielded an overall test accuracy of 85.7%. However, their model relied on a more sophisticated neural network–based approach and additionally required diffusion-weighted imaging as input modality, thereby potentially limiting clinical applicability.

Our study has some limitations. First, we restrict ourselves to the anatomical MRI sequences and do not investigate functional MRI sequences such as diffusion and perfusion which may allow to further improve the performance when predicting molecular glioma subtypes [29, 30]. However, in contrast to anatomical sequences, functional sequences are not represented in arbitrary but absolute values and are thus comparable, even though the influence of normalization on functional MRI sequences should be part of further research. Second, we did not perform hyperparameter tuning, e.g., through grid search which may allow to improve the model performance, but instead kept the model parameters constant to eliminate potential bias when assessing the model performance of different sequence normalization approaches. Third, both *Z*-score and WhiteStripe normalization are quite similar methods, and we did not compare novel ones such as convolutional neural network–based methods [31]. However, as these two methods are among the most widely used in radiomic research [32] and there are hardly any publications that compare two or more standardization methods, this study provides a valid basis and the comparison to novel methods needs to be explored in the future.

In conclusion, in this study we were able to develop a reliable multiclass model for the classification of glioma genetic subtypes and demonstrate the relevance of intensity normalization of MR images to increase the generalizability of this models.

### Supplementary information

Below is the link to the electronic supplementary material.

Supplementary file1 (RAR 1116 KB)

## Abbreviations

CNN: Convolutional neural network
IBSI: Image Biomarker Standardisation Initiative
IDH: Isocitrate dehydrogenase
NAWM: Normal-appearing white matter
ROI: Region of Interest
RQS: Radiomics Quality Score
TCGA: The Cancer Genome Atlas
TRIPOD: Transparent reporting of a multivariable prediction model for individual prognosis or diagnosis
UCSF: University of California San Francisco
WHO: World Health Organization

## Declarations

### Guarantor

The scientific guarantor of this publication is Philipp Vollmuth.

### Statistics and biometry

One of the authors (PV and MFD) has significant statistical expertise.

### Study subjects or cohorts overlap

No study subjects or cohorts have been previously reported.

### Methodology

- retrospective
- diagnostic or prognostic study
- multicenter study
